# MicroRNA signatures in inflammatory dental tissues: biomarkers and molecular mechanisms

**DOI:** 10.3389/fmolb.2026.1881450

**Published:** 2026-08-18

**Authors:** Dandan Shao, Zhenyu Yao

**Affiliations:** 1 The First Affiliated Hospital, College of Clinical Medicine, Henan University of Science and Technology, Luoyang, Henan, China; 2 Sanya Hospital of Traditional Chinese Medicine (Hainan Hospital, Guangzhou University of Chinese Medicine), Clinical Medicine Research Center, Sanya, Hainan, China

**Keywords:** extracellular vesicles, immune regulation, MicroRNAs, pulpitis, tissue regeneration

## Abstract

Inflammatory immune responses in the dental pulp are shaped by interacting cellular and molecular processes, and microRNAs (miRNAs) are important post-transcriptional regulators. However, an integrated understanding of how miRNA networks orchestrate the balance between pulpitis progression and tissue repair remains incomplete. This review examines the differential expression profiles of miRNAs in inflamed dental pulp, and evaluates their potential as adjunctive biomarkers of pulpitis status. We also summarize how miRNAs regulate key inflammatory signaling cascades, including the TLR/NF-κB, mitogen-activated protein kinase, and cGAS-STING pathways, and discuss their integration within broader epigenetic crosstalk networks involving lncRNAs and circRNAs. We further analyze how the inflammatory microenvironment and physicochemical stresses alter the osteogenic/odontogenic differentiation potential of dental pulp stem cells through miRNA-dependent regulation. We then assess the translational prospects of exosome-delivered miRNAs as cell-free therapeutics and highlight unresolved challenges in sampling feasibility, donor heterogeneity, delivery specificity, and clinical validation. By synthesizing evidence on miRNA regulatory networks in pulpal inflammation and regeneration, this review provides a framework for evaluating miRNA-based diagnostic tools and targeted therapies for pulpitis.

## 1 Introduction

Pulpitis, most often triggered by carious infection, initiates an inflammatory immune response that integrates cellular and humoral immunity with local neurovascular changes to eliminate invading pathogens. If pathogen clearance is incomplete, inflammation may progress to irreversible pulp necrosis (Väisänen et al., 2025).

MicroRNAs (miRNAs), short noncoding RNAs approximately 19–23 nucleotides long, have become central to research on this pathological process. Since their discovery, miRNAs regulate inflammatory responses (Maqbool et al., 2023). They typically repress gene expression post-transcriptionally by binding to the 3′ untranslated region (3′UTR) of target messenger RNAs (mRNAs), through sequence-dependent interactions (Tavares et al., 2015). The miRNA regulatory network is extensive, with reported involvement in the regulation of more than 60% of human genes, as well as cell self-renewal, proliferation, apoptosis, and immunity (Soheilifar et al., 2023). In the complex network of competing endogenous RNAs, miRNAs act as post-transcriptional modulators, directly influencing the differentiation of odontoblasts and the progression of related diseases (Sun et al., 2015).

In the pulp microenvironment, small changes in non-coding RNA expression can alter cell fate (Zeng and Huang, 2022). miRNAs regulate physiological and pathological processes in dental pulp, including inflammation, and influence the maintenance of stemness and differentiation potential of dental pulp stem cells (DPSCs) (Kulthanaamondhita et al., 2022). Therefore, mesenchymal stem cells derived from human dental pulp tissue are considered a promising cell source for regenerative therapy, and a detailed understanding of the miRNA networks that regulate their directed differentiation is a prerequisite for clinical translation (Iranmanesh et al., 2023).

Exosomes are natural nanoscale extracellular vesicles of interest for cell-free therapy because they carry bioactive cargo, including miRNAs (Shi et al., 2020). Exosomes derived from dental pulp stem cells carry bioactive cargo that can alter the dental pulp microenvironment through paracrine mechanisms and promotes oral and maxillofacial tissue regeneration, and may reduce the risk of immune rejection associated with direct stem cell transplantation (Mottaghi et al., 2025). Comparing miRNA composition in dental pulp tissue and exosomes in both healthy and inflammatory states may clarify disease-associated mechanisms and inform regenerative strategies (Chansaenroj et al., 2025).

Although previous reviews have summarized the general biological functions of miRNAs in dental pulp stem cells, the dynamic interplay between miRNA-mediated post-transcriptional regulation and the evolving inflammatory microenvironment has not been integrated systematically. To address this gap, this review synthesizes current evidence of the differential expression profiles of miRNAs in pulpitis and examines their molecular mechanisms in immune modulation. We also focus on the effects of inflammatory and physicochemical stress on the differentiation potential of dental pulp cells, and evaluate translational prospects and current limitations of exosome-delivered miRNAs. By integrating these lines of evidence, this narrative review complements existing literature and provides a focused framework for the development of miRNA-based diagnostic tools and cell-free regenerative endodontic therapies.

## 2 Literature search strategy

To provide a comprehensive narrative overview, we searched PubMed and Web of Science. The search strategy used combinations of the following keywords: (“microRNA” OR “miRNA”) AND (“pulpitis” OR “dental pulp inflammation” OR “dental pulp stem cells” OR “DPSC” OR “exosomes” OR “extracellular vesicles” OR “biomarker” OR “immune regulation” OR “tissue regeneration”). We included only peer-reviewed articles published in English. Because this was not a systematic review, we selected studies for their scientific relevance and contribution to the review framework. We prioritized studies that investigated miRNA expression profiles in dental tissues, inflammatory signaling cascades, DPSC differentiation, and their translational applications in endodontics. Because this was a narrative review, we did not apply formal quality-assessment tools or PRISMA guidelines. Evidence was synthesized narratively to connect miRNA expression profiles, inflammatory mechanisms, DPSC differentiation and repair-oriented translation.

## 3 Differential expression profiles and biomarker potential of miRNAs in pulpitis

**FIGURE 1:**
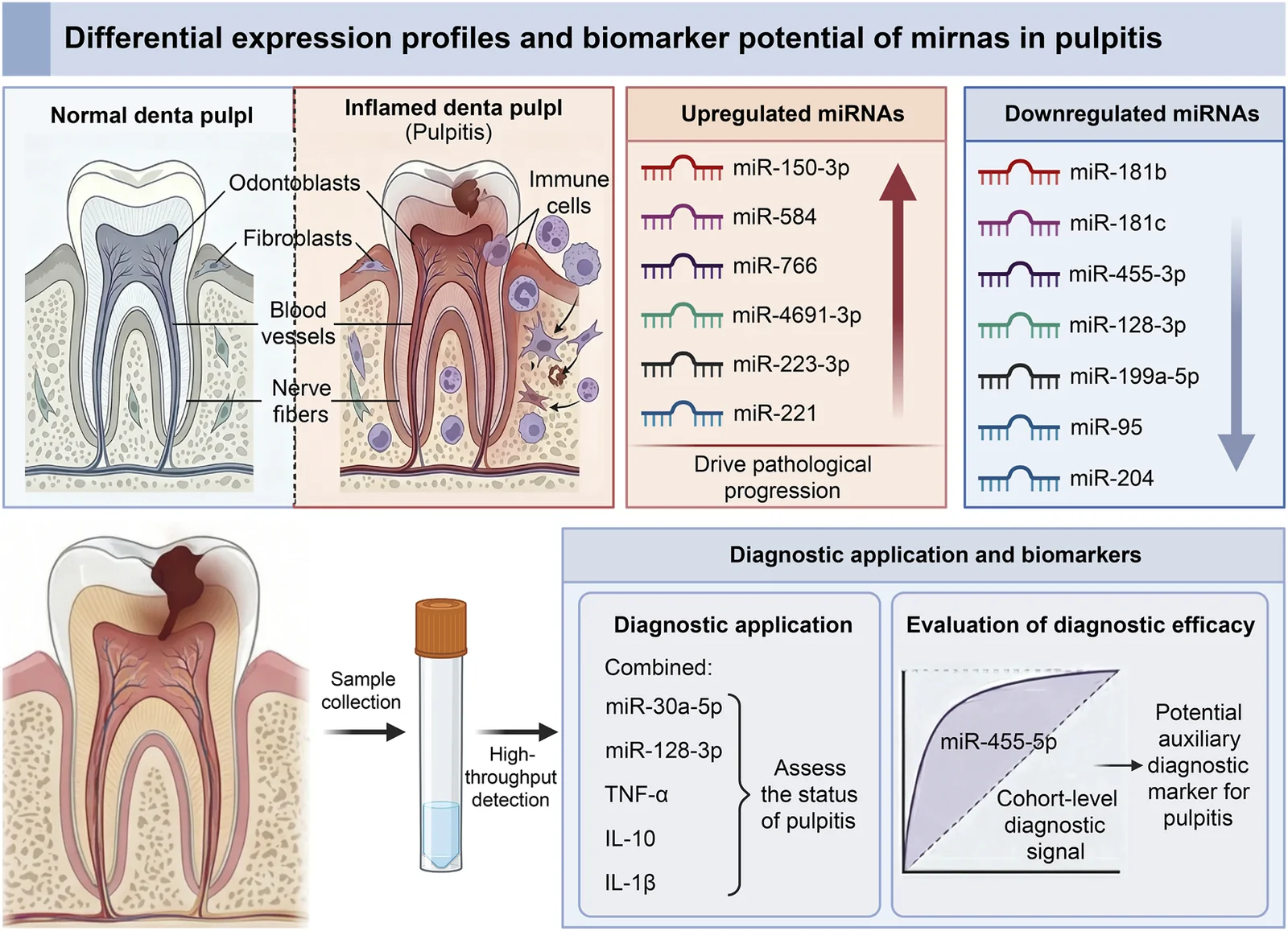
Differential expression profiles and biomarker potential of miRNAs in pulpitis. The schematic compares normal and inflamed dental pulp and shows immune-cell infiltration and associated microenvironmental changes. Inflammation is accompanied by remodeling of the miRNA expression profile. Reported increases include miR-150-3p, miR-584, miR-766, miR-4691-3p, miR-223-3p, and miR-221, whereas decreases include miR-181b/c, miR-455-3p, miR-128-3p, miR-199a-5p, miR-95, and miR-204. The lower panel summarizes the diagnostic potential of differentially expressed miRNAs. After sample collection and high-throughput profiling, molecular signatures may support objective assessment. The combined detection of miR-30a-5p and miR-128-3p alongside inflammatory cytokines TNF-α, IL-10, and IL-1β has been proposed to assess pulpitis status. In the reported cohort, ROC analysis identified a diagnostic signal for miR-455-5p, although clinical validation remains necessary. (Figure created using BioRender.com).

Infection and inflammatory stress markedly remodel the post-transcriptional regulatory network of pulp tissue. High-throughput screening and microarray studies have mapped the differential expression of miRNAs between normal and inflamed dental pulp tissues (Zhong et al., 2012). Here, “abnormally expressed miRNAs” denotes miRNAs reported as significantly upregulated or downregulated in pulpitis, apical periodontitis, or matched experimental inflammatory models; these molecules may contribute to disease progression and serve as candidate adjunctive biomarkers for clinical diagnosis (Figure 1).

### 3.1 Abnormally elevated miRNAs during inflammation

High-throughput profiling of human dental pulp tissue has identified multiple differentially expressed miRNAs (Table 1). An early profiling study found that miR-150-3p, miR-584, and miR-766 were significantly elevated in inflamed pulp among 335 detected miRNAs, suggesting a potential role in pulp immune responses (Zhong et al., 2012). Subsequent work further confirmed high miR-584-5p expression in irreversible pulpitis (IP) tissues (Zou et al., 2026). miR-4691-3p was significantly increased in IP samples and could be further induced by pro-inflammatory factors such as TNF-α and IL-6, suggesting sensitivity to inflammatory stimulation (Tian X. et al., 2023). As a potential participant in compensatory repair, miR-223-3p was also significantly upregulated in the inflamed pulp (Huang et al., 2019). *In vitro*, transcription-factor perturbation can also remodel miRNA expression. For example, Oct-4B1 deletion induced a significant increase in miR-221 in stimulated pulp cells, which in turn regulates a complex downstream signaling network (Kong et al., 2014).

**TABLE 1:** Differential miRNA expression profiles and biomarker potential in pulpitis.

Classification	Core molecules	Expression trend in pulpitis	Biological finding and diagnostic relevance	Evidence type	References
Abnormally elevated miRNAs under inflammation	miR-150, miR-584, miR-766	Upregulated	Elevated in inflamed pulp and associated with pulpal immune responses	Human tissue	Zhong et al. (2012)
	miR-584-5p	Upregulated	Highly expressed in irreversible pulpitis (IP) tissue	Human tissue/*in vitro*	Zou et al. (2026)
	miR-4691-3p	Upregulated	Induced by pro-inflammatory cytokines, including TNF-α and IL-6, in the reported model	*In vitro*	Tian et al. (2023b)
	miR-223-3p	Upregulated	Upregulated in inflamed pulp and associated with compensatory tissue repair	Human tissue/*in vitro*	Huang et al. (2019)
	miR-221	Upregulated	Elevated after cellular stimulation and Oct-4B1 depletion, with accompanying signaling changes	*In vitro*	Kong et al. (2014)
Predominantly downregulated miRNAs during disease progression	miR-181b, 181c, 455-3p, 128-3p, 199a-5p, 95	Downregulated	Reported as consistently downregulated in pulpal or periapical lesions	Systematic review	Al Gashaamy et al. (2023)
	Set of 24 differentially expressed miRNAs	Downregulated	Predicted to target IL-6, MMP-9, TGF-β, and related mediators of progression to periapical disease	Human tissue/bioinformatics	Chan et al. (2013)
	miR-181b	Downregulated	Reduced in damaged tissue; restoration suppressed inflammatory cytokines in cell and animal models	*In vitro*/animal model	Meng et al. (2024)
	miR-204	Downregulated	Reduced in pediatric pulpitis with *H. pylori* coinfection and associated with increased MMP9 protein	Human tissue	Zhou and Xu (2019)
Potential adjunctive diagnostic indicators	miR-30a-5p, miR-128-3p	Downregulated	Combined measurement with TNF-α, IL-10, and IL-1β has been proposed for objective assessment of vital-pulp inflammation	Human tissue	Louzada et al. (2024)
	miR-455-5p	Downregulated	ROC analysis suggests diagnostic discrimination in the reported cohort; further validation is needed before clinical use	Human tissue/*in vitro*	Hu et al. (2025)
	Fibroblast-derived exosomal miRNAs	—	Display MSC-like molecular signatures, supporting evaluation of minimally invasive body-fluid monitoring	*In vitro*	Yoshida et al. (2026)
	Immune response proteins, Substance P	Upregulated	Proteomic and neuropeptide changes associated with local inflammation and stress responses	Human tissue	Caviedes-Bucheli et al. (2009), Silva et al. (2021)

### 3.2 Broad miRNA downregulation during disease progression

In the microenvironment of pulpitis, broad miRNA downregulation appears to be common and pathologically relevant, often resulting in disinhibition of pro-inflammatory target genes. A systematic review by Al Gashaamy et al. reported that 40 of the 44 differentially expressed miRNAs associated with pulpitis were downregulated (Al Gashaamy et al., 2023). In particular, hsa-miR-181b, hsa-miR-181c, hsa-miR-455-3p, hsa-miR-128-3p, hsa-miR-199a-5p, and hsa-miR-95 showed consistent downregulation in lesional tissue (Al Gashaamy et al., 2023). Chan et al. compared diseased pulp/periapical tissues with control tissues and identified a network of 24 downregulated miRNAs (Chan et al., 2013). Their bioinformatic analysis suggested that these miRNAs may target immune and matrix-remodeling mediators such as IL-6, MMP-9, and TGF-β, thereby linking miRNA downregulation to the progression from pulpal inflammation to periapical disease. Meng et al. further showed that miR-181b was reduced in damaged human dental pulp tissue and that restoration of miR-181b suppressed PLAU/AKT/NF-κB signaling, inflammatory cytokine production and pulp destruction in cell and animal models (Meng et al., 2024). These findings indicate that miRNA downregulation may remove post-transcriptional constraints on inflammatory effector pathways. In a specific clinical setting, children with pulpitis complicated with *Helicobacter pylori* infection, showed reduced pulpal miR-204 expression associated with increased matrix metalloproteinase 9 (MMP9) (Zhou and Xu, 2019).

### 3.3 Potential of miRNAs as adjunctive diagnostic markers

Because assessment of pulpitis status relies heavily on subjective clinical symptoms, molecular diagnostic tools based on stable miRNA signals and their relationship to inflammation severity could be clinically useful. One clinical tissue study found that the expression of miR-30a-5p and miR-128-3p was significantly reduced in irreversible pulpitis and in vital pulp tissues with severe periodontal damage. Combined measurement of these miRNAs and TNF-α, IL-10 and IL-1β has been proposed as an objective approach to assessing pulpitis status (Louzada et al., 2024). In diagnostic-performance analyses, miR-455-5p showed discriminatory performance in the reported cohort by receiver operating characteristic (ROC) analysis and may represent a candidate adjunctive diagnostic marker for pulpitis (Hu et al., 2025).

Dental pulp-derived fibroblasts secrete exosomal miRNAs with molecular features similar to those of mesenchymal stem cells, providing a rationale for minimally invasive monitoring using exosomes (Yoshida et al., 2026). Proteomic analyses support broader molecular changes: inflamed pulp is enriched in proteins associated with immune responses, platelet activation, and cellular stress (Silva et al., 2021). Substance P levels also vary with anesthetic and vasoconstrictor conditions, reflecting the complex local inflammatory microenvironment (Caviedes-Bucheli et al., 2009).

Clinical translation of miRNA-based diagnosis remains constrained by sampling feasibility. Most current evidence relies on extracted, exposed or otherwise accessible pulp-related tissues; therefore, applying miRNA profiling to clinically closed reversible pulpitis would require validated minimally invasive sample sources and standardized thresholds before routine chairside use.

## 4 Core molecular mechanisms by which miRNAs regulate the immune response in pulpitis

**FIGURE 2:**
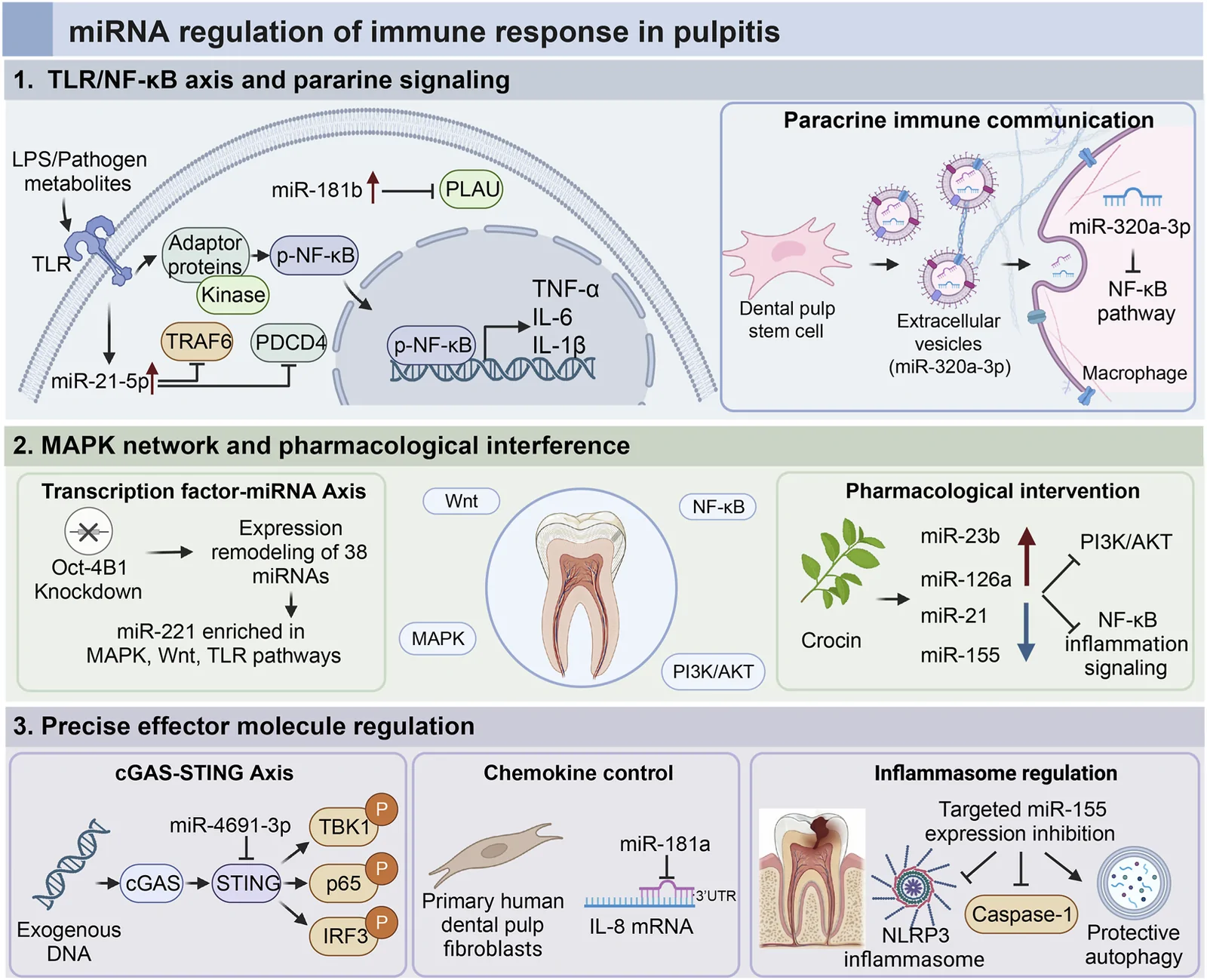
Core molecular mechanisms of miRNA-mediated immune regulation in pulpitis. The TLR/NF-κB and paracrine-signaling module shows how intracellular miR-21-5p and miR-181b, together with exosome-delivered miR-320a-3p, restrain inflammatory signaling and support intercellular communication. The MAPK module depicts crosstalk among Oct-4B1, miRNAs, and related pathways, as well as the effects of crocin-associated miRNA changes. The effector module summarizes miRNA-mediated regulation of cGAS-STING signaling, IL-8 translation, and NLRP3/caspase-1 activation in relation to inflammation and autophagy. (Figure created using BioRender.com).

When pathogenic microorganisms and their metabolites invade pulp tissue, cell-surface pattern-recognition receptors (PRRs) are rapidly activated, triggering complex intracellular signaling cascades. Within this innate immune network, miRNAs act as key post-transcriptional mediators, balancing inflammatory amplification against excessive tissue damage by binding to specific target genes (Figure 2).

### 4.1 Post-transcriptional regulation of the canonical TLR/NF-κB signaling axis

Toll-like receptors (TLRs) and their downstream nuclear factor-κB (NF-κB) signaling pathways form a central axis mediating dental pulp immune responses. Several miRNAs negatively regulate this axis by targeting its adaptor proteins. In lipopolysaccharide (LPS)-stimulated human dental pulp cells, the canonical TLR/NF-κB pathway is activated along with compensatory upregulation of miR-21-5p. Overexpression of the miRNA establishes a negative-feedback loop by directly downregulating tumor necrosis factor receptor-associated factor 6 (TRAF6) and programmed cell death protein 4 (PDCD4), thereby inhibiting the release of downstream pro-inflammatory cytokines (Nara et al., 2019).

Similarly, by directly targeting urokinase-type plasminogen activator (PLAU), miR-181b inhibits AKT/NF-κB signaling and reduces LPS-induced pulp destruction in cell and animal models (Meng et al., 2024). In addition to cell-intrinsic regulation, miRNAs are also involved in intercellular immune communication. For example, inflammation-preconditioned dental pulp stem cells can secrete extracellular vesicles enriched in the anti-inflammatory miRNA miR-320a-3p. After being endocytosed by macrophages, these vesicles modulate the NF-κB pathway of target cells, supporting a paracrine immunomodulatory role (Umar et al., 2024).

### 4.2 Regulation of MAPK networks and pharmacological targets

In the inflammatory microenvironment, the mitogen-activated protein kinase (MAPK) pathway shows extensive crosstalk with other signaling networks such as Wnt and PI3K. *In vitro* and bioinformatic studies suggest that the interaction between transcription factors and miRNAs operates at this multipathway interface. Knockdown of oct-polymer-binding transcription factor 4B1 (Oct-4B1) in LPS-treated dental pulp cells altered the expression of 38 miRNAs. miR-221 was among the affected transcripts, and bioinformatic analysis predicted enrichment of its targets in MAPK, Wnt and Toll-like receptor pathways, supporting a role for the transcription factor-miRNA axis in shaping the pulpitis cascade (Kong et al., 2014).

This miRNA-based multipathway regulation also provides a rationale for evaluating anti-inflammatory drugs. For example, treatment of dental pulp mesenchymal stem cells with the plant-derived bioactive compound crocin alters the intracellular miRNA expression profile. Specifically, crocin upregulates miR-23b and miR-126a while downregulating miR-21 and miR-155. This coordinated miRNA profile was associated with reduced activity in the PI3K/Akt and NF-κB inflammatory signaling pathways and enhanced immunomodulatory activity in the reported model (Rostami et al., 2024).

### 4.3 Regulation of chemokines, cGAS-STING and inflammasomes

At the effector level, specific miRNAs have been reported to directly silence pathways involved in chemokine synthesis, exogenous nucleic acid recognition and inflammasome assembly. During responses to exogenous DNA, miR-4691-3p negatively regulates the cGAS-STING signaling cascade by directly binding to and inhibiting STING, thereby blocking the phosphorylation of TBK1, p65 and IRF3 and downstream expression of IFN-β, TNF-α and IL-6, thereby nominating this axis as a candidate target in deep pulp inflammation (Tian X. et al., 2023).

miR-181a was first identified as a direct post-transcriptional regulator of interleukin-8 (IL-8) of neutrophil chemotaxis. In TLR4/2-positive primary human dental pulp fibroblasts exposed to Porphyromonas gingivalis LPS, miR-181a limits IL-8 translation by directly binding to the 3′UTR of the IL-8 transcript (Galicia et al., 2014). In addition, miRNAs may contribute to regulating the balance between pyroptosis and autophagy. In pulpitis models, miR-155 inhibition attenuates local inflammatory responses and induces protective autophagy by reducing expression of nucleotide-binding oligomerization domain-like receptor protein 3 (NLRP3) inflammasome and caspase-1.

## 5 Epigenetic and ceRNA crosstalk networks in the microenvironment of pulpitis

In the pathological microenvironment of pulpitis, gene expression is not controlled by a single linear miRNA pathway. Instead, a ceRNA network integrates DNA methylation, chromatin remodeling, diverse lncRNAs and circRNAs (Table 2).

**TABLE 2:** Epigenetic and ceRNA crosstalk networks in the pulpitis microenvironment.

Classification	Core regulators	Targeted miRNA	Downstream targets or signals	Reported biological effect or outcome	Evidence type	Reference
DNA methylation and transcriptional remodeling	DNMT1	miR-146a-5p	—	Contributes to regulation of the LPS-induced inflammatory cascade in pulpitis	*In vitro*	Mo et al. (2019)
	lncRNA chr22-38	miR-125b-5p	STAT3	Predicted to affect STAT3-linked chromatin remodeling and disease progression	Bioinformatic analysis	Li and Sun (2025)
lncRNA-mediated inflammatory exacerbation	CARD8-AS1, LINC00924	Specific immune-related miRNAs	JAK-STAT, TLR pathways	Identified as candidate immune predictors linked to pulpal inflammatory pathways	Bioinformatic analysis	Xu et al. (2025)
	TFAP2A-AS1	miR-32-5p	—	Promotes pro-inflammatory cytokine release and impairs odontogenic potential	Human tissue/*in vitro*	Liu et al. (2024b)
lncRNA-mediated stem cell differentiation	G043225	miR-588	FBN1	Maintains the cellular phenotype through a ceRNA network	*In vitro*/bioinformatics	Chen et al. (2020b)
	CCAT1	miR-218	—	Promotes the proliferation and differentiation of dental pulp stem cells (DPSCs)	*In vitro*	Zhong et al. (2019)
	DLX6-AS1	miR-128-3p	MAPK14 axis	Promotes stem-cell differentiation toward osteogenic and odontogenic lineages	*In vitro*	Wu et al. (2024a)
	IGFBP7-AS1	miR-335-3p, miR-155-5p	—	Enhances odontogenic differentiation through a multi-miRNA interaction network	*In vitro*	Zhu et al. (2023)
circRNA sponging in inflammation and differentiation	hsa_circ_0001978, hsa_circ_0004417	Associated miRNAs	MAPK, Wnt pathways	May activate inflammatory pathways and amplify DPSC inflammatory responses	*In vitro*	Lei et al. (2022)
	Differentiation-associated circRNA profiles	—	—	Show extensive changes during odontogenic differentiation	*In vitro*	Chen et al. (2020a)
	circKLF4	miR-1895, miR-5046	Klf4	Upregulates Klf4 through interactions with miR-1895 and miR-5046 during differentiation	Animal-derived cell model	Zhang et al. (2021)
	hsa_circ_0026827	miR-188-3p	—	Promotes osteoblast differentiation	*In vitro*	Ji et al. (2020)
	circRNA124534	—	—	Reported to promote osteogenic differentiation	*In vitro*	Ji et al. (2020)
Cross-species regulation and microenvironmental subversion	Exogenous viral vmiRs	Target host mRNAs	Host chemokines, immune signaling molecules	May interfere with host chemotactic and immune signaling across species	Human tissue	Zhong et al. (2017)

### 5.1 DNA methylation, chromatin remodeling and transcriptional hierarchy of lncRNAs

Epigenetic modifications can directly reshape the miRNA expression profile under inflammatory stress. DNA methyltransferase 1 (DNMT1) expression decreased under lipopolysaccharide (LPS) stimulation, and miR-146a-5p was significantly upregulated after methylation-inhibitor treatment, indicating that DNA methylation contributes to the regulation of the inflammatory cascade (Mo et al., 2019). At a broader transcriptional-regulatory level, high-throughput sequencing revealed differential expression and complex interaction networks among lncRNAs, miRNAs, and mRNAs in inflamed dental pulp (Liu et al., 2020). Bioinformatic analysis further identified STAT3 as a core gene associated with chromatin remodeling in pulpitis and revealed that lncRNA chr22-38_28785274-29006793.1 may upregulate STAT3 by “sponging” miR-125b-5p, potentially contributing to disease progression (Li and Sun, 2025).

### 5.2 LncRNA-mediated inflammatory amplification and differentiation networks

In bioinformatic and cell-based ceRNA analyses, lncRNAs may influence immunity and differentiation, although the strength of evidence varies. In immune-related prediction models, immune-related lncRNAs such as CARD8-AS1 and LINC00924 were identified as key predictors of pulpitis, and were linked to JAK-STAT and TLR pathway regulation through interactions with specific miRNAs (Xu et al., 2025). For inflammatory amplification, LPS-induced TFAP2A-AS1 upregulation promoted pro-inflammatory cytokine release through competitive binding to miR-32-5p and impaired odontogenic potential, whereas TFAP2A-AS1 silencing reversed these inflammatory and differentiation-related effects (Liu M. et al., 2024).

Beyond inflammation, several studies have examined the role of lncRNAs in regulating osteogenic/odontogenic differentiation of dental pulp stem cells. Whole-genome screening was used to construct a ceRNA network centered on lncRNA G043225, suggesting that it regulates FBN1 expression by binding to miR-588 to maintain the cell phenotype (Chen Z. et al., 2020). In specific differentiation-promoting pathways, lncRNA CCAT1 promotes the proliferation and differentiation of dental pulp stem cells by negatively regulating miR-218 (Zhong et al., 2019). lncRNA DLX6-AS1 promotes odontogenic/osteogenic differentiation by targeting the miR-128-3p/MAPK14 axis (Wu B. et al., 2024). IGFBP7-AS1 has been shown to enhance odontogenic differentiation through an interaction network with miR-335-3p and miR-155-5p (Zhu et al., 2023).

### 5.3 CircRNA sponging and cross-species viral vmiR interference

CircRNAs, through their stable covalently closed structures, can also act as potent miRNA sponges. In the inflammatory microenvironment, TNF-α alters circRNA expression profiles in dental pulp stem cells, and upregulated hsa_circ_0001978 and hsa_circ_0004417 may indirectly activate the MAPK and Wnt pathways by sequestering miRNAs, potentially amplifying inflammation (Lei et al., 2022).

Evidence for differentiation-related circRNAs is more extensive. Expression profiling has identified numerous circRNAs that change during odontoblast differentiation (Chen M. et al., 2020). Specifically, circKLF4 can upregulate the expression of Klf4 by sponging miR-1895 and miR-5046 (Zhang et al., 2021), hsa_circ_0026827 promotes osteoblast differentiation by sequestering miR-188-3p (Ji et al., 2020), and circRNA124534 has also been reported to promote osteogenic differentiation (Ji et al., 2020).

Finally, dental pulp regulation may also be influenced by cross-species viral miRNAs. One human study detected 12 virus-encoded microRNAs (vmiRs) were detected in dental pulp, four of which were differentially expressed in inflamed tissue. Bioinformatic predictions suggested that pathogen-derived vmiRs, including herpesvirus miRNAs, may disrupt dental-pulp defense by targeting host chemotactic and immune signaling molecules (Zhong et al., 2017).

## 6 Tissue damage in an inflammatory microenvironment and pulp repair

Dental pulp stem cells (DPSCs) within dental pulp tissue retain capacity for tissue repair and multilineage differentiation when exposed to pathogen invasion or external stimuli. In this pathophysiological transition from destruction to repair, miRNAs form post-transcriptional hubs that regulate stem cell proliferation, apoptosis, and lineage commitment. Their functions depend on intrinsic cell state and are further remodeled by the local physicochemical microenvironment.

### 6.1 Opposing effects of inflammation, physicochemical stress and toxin exposure on differentiation potential

In the pathological microenvironment, inflammatory factors and stress conditions have opposing, context-dependent effects on stem cell differentiation (Table 3). As a compensatory response, the LPS-induced microenvironment promotes overexpression of miR-223-3p, which promotes odontoblast differentiation by specifically reducing SMAD3 protein levels (Huang et al., 2019). Extracellular vesicles released under inflammatory conditions have also been shown to carry specific non-coding RNA cargo, which can alter the osteogenic and odontogenic potential of recipient cells (Yan et al., 2022). Conversely, inflammatory signaling can impair differentiation. For example, upregulated miR-584-5p exacerbates tissue inflammation by inhibiting PTEN and impairs osteogenic differentiation (Zou et al., 2026), while miR-140-5p was also identified as an inhibitor of proliferation and differentiation in LPS-mediated models (Sun et al., 2017).

**TABLE 3:** Tissue repair in the pathological microenvironment and miRNA regulatory networks.

Classification	Core regulators	Expression trend in pulpitis	Target genes or signals	Reported biological effect or outcome	Evidence type	Reference
Inflammation and physicochemical/toxic stress	miR-223-3p	Upregulated	SMAD3	Promotes odontoblast differentiation by reducing SMAD3	Human tissue/*in vitro*	Huang et al. (2019)
	Inflammatory extracellular vesicles (EVs)	Upregulated	—	Alter the osteogenic and odontogenic potential of recipient cells	*In vitro*/animal model	Yan et al. (2022)
	miR-584-5p	Upregulated	PTEN	Exacerbates tissue inflammation and hinders osteogenic differentiation	Human tissue/*in vitro*	Zou et al. (2026)
	miR-140-5p	Upregulated	—	Inhibits proliferation and differentiation	*In vitro*	Sun et al. (2017)
	Hypoxia/Hypoxic EVs	—	—	Remodels RNA profiles and enhances cell survival; hypoxic EVs improved bone repair in animal models	*In vitro*/animal model	Shi et al. (2019), Tian et al. (2023a)
	Mechanical stress	—	—	Alters miRNA expression during osteogenic and odontogenic differentiation	*In vitro*	He et al. (2021)
	Lead toxin/Nicotine (Smoking)	—	p53 pathway	Induces miRNA changes associated with impaired stem-cell reparative capacity	*In vitro*	Hardin et al. (2024), Khalid et al. (2022), Vang et al. (2025)
Differentiation-promoting factors	miR-93-5p	—	KDM6B	Directly promotes dentin formation	*In vitro*/animal model	Wu et al. (2024b)
	miR-27a	—	DKK3, SOSTDC1	Relieves inhibition of mineralization-related signaling	*In vitro*/animal model	Yu et al. (2025)
	miR-146a-5p	Upregulated	Notch1	Promotes osteogenic lineage commitment through Notch1-related signaling	*In vitro*	Yu et al. (2022), Qiu et al. (2019)
	miR-483-3p	—	—	Promotes osteogenic differentiation *in vitro*	*In vitro*	Yu et al. (2024)
	Lin28	—	let/7b	Blocks let-7b precursor maturation and relieves translational repression of osteogenic genes	*In vitro*	Liu et al. (2019b), Yan et al. (2024)
	FOXA1 (↓)/SUV39H1 (↑)	—	—	Associated with increased mineralization potential of stem cells	*In vitro*	Li et al. (2022), Wang et al. (2025)
	miR-218	—	—	Contributes to the multilineage differentiation potential of stem cells	*In vitro*	Gay et al. (2014)
Differentiation restraints and early fate	miR-720	—	—	Helps maintain the undifferentiated stem-cell phenotype	*In vitro*	Hara et al. (2013)
	miR-145, miR-143	—	Klf4, Osx	Restricts excessive odontoblast maturation	Animal-derived cell model	Liu et al. (2013)
	miR-508/342/6807-5p	—	—	Inhibit osteogenic and odontogenic potential at specific differentiation stages	*In vitro*	Liu et al. (2019a), Wang et al. (2021), Zeng et al. (2023)
	miR-135b	—	—	Inhibits osteogenic and odontogenic potential	*In vitro*	Song et al. (2017)
	miR-15b-5p, miR-206-3p	—	IGF1	Associated with early odontogenic development	Developmental tissue/cell model	Neupane et al. (2020)
Angiogenesis and soft-tissue differentiation	miR-424	—	—	Promotes angiogenesis *in vitro*	*In vitro*	Liu et al. (2014)
	Periodontitis-associated EVs containing miR-378a	Upregulated	Sufu → Gli1	Increased secretion under periodontitis-associated conditions promotes local vascularization	*In vitro*/animal model	Zhou et al. (2021)
	miR-584	Upregulated	—	Knockdown promotes proliferation	*In vitro*	Tian et al. (2020)
	miR-224-5p	Downregulated	Rac1	Downregulation promotes migration and proliferation, whereas expression protects against apoptosis	*In vitro*	Ke et al. (2019), Qiao et al. (2020)
	miR-139-5p	—	—	Provides indirect soft-tissue lineage evidence for DPSC plasticity	*In vitro*	Xie and Shen (2018)
	miR-20a-5p	—	SMAD6	Provides indirect chondrogenic-lineage evidence for DPSC plasticity	*In vitro*	Pan et al. (2023)
Aging and tissue engineering interventions	Aging-signature miRNAs	—	—	Associated with expression differences and reduced cellular repair potential	Human tissue/*in vitro*	Iezzi et al. (2021), Wang et al. (2015)
	Inorganic interfaces (Portland cement, modified Ti, and related materials)	Upregulated	miR-146a	Enhance osteogenic attachment and differentiation of cells at the interface	*In vitro*/biomaterial model	Gardin et al. (2016), Tumedei et al. (2022), Wang et al. (2018)
	Engineered or lineage-specific exosomes	—	—	May address limitations of cell-based approaches and guide stem-cell differentiation	*In vitro*/animal model	Hu et al. (2019), Zhang et al. (2024)

Beyond inflammatory signals, physicochemical stressors can reshape miRNA expression patterns. Hypoxia preconditioning can globally reshape the coding and noncoding RNA profiles of DPSCs, thereby enhancing cell survival under ischemic conditions (Shi et al., 2019). Small extracellular vesicles derived from stem cells under hypoxic conditions have reduced inflammatory osteolysis and promoted deep bone repair (Tian J. et al., 2023). Mechanical stress can also alter miRNA expression substantially during osteogenic/odontogenic differentiation (He et al., 2021). Among environmental and lifestyle exposures, lead exposure impairs the odontogenic potential of the dental pulp by remodeling the molecular network (Khalid et al., 2022). Nicotine induces a distinct miRNA signature (Vang et al., 2025), whereas other tobacco-related compounds impair the reparative capacity of dental pulp stem cells through the miRNA-mediated p53 pathway (Hardin et al., 2024).

### 6.2 MiRNA regulatory networks for osteogenic and odontogenic differentiation

Complex post-transcriptional networks drive key phenotypic transformations during the lineage commitment of stem cells to osteoblasts or odontoblasts. Genome-wide microarray and transcriptomic studies have characterized miRNA dysregulation during dental-pulp-cell differentiation (Aung et al., 2020), identified regulators of dentin sialophosphoprotein (DSPP) (Huang et al., 2011), and described transcriptional changes during epigenetic reprogramming and mineralization processes (Fujii et al., 2024; Gong et al., 2012; Kearney et al., 2023). Among differentiation-promoting miRNAs, miR-93-5p directly promotes dentin formation by targeting histone demethylase KDM6B (Wu S. et al., 2024); miR-27a synergistically inhibits DKK3 and SOSTDC1 to activate mineralization-related signaling (Yu et al., 2025), whereas miR-483-3p (Yu et al., 2024) and miR-146a-5p (Yu et al., 2022) promote osteogenic differentiation in the reported *in vitro* differentiation systems. The transcription of these miRNAs is also regulated by upstream factors. For example, the RNA-binding protein Lin28 relieves the translational inhibition of proliferative and osteogenic genes by blocking the maturation of let-7b precursors (Liu Y. et al., 2019; Yan et al., 2024). Knockdown of the transcription factor FOXA1 (Li et al., 2022) and upregulation of histone methyltransferase SUV39H1 (Wang et al., 2025), and activation of Notch-related axes, including the miR-146a-5p/Notch1 axis (Kulthanaamondhita et al., 2024; Qiu et al., 2019), have each been associated with increased mineralization potential in stem cells. miR-218 also contributes to the multilineage differentiation potential of stem cells (Gay et al., 2014).

To maintain the homeostasis of the stem-cell pool and prevent pathological ectopic calcification, post-transcriptional constraints preserve this balance. In the undifferentiated state, miR-720 helps maintain stem cell phenotypes (Hara et al., 2013). As differentiation progresses, miR-145 and miR-143 jointly target the rate-limiting transcription factors Klf4 and Osx to limit overmaturation of odontoblasts (Liu et al., 2013). In addition, miR-508-5p (Liu F. et al., 2019), miR-342-5p (Zeng et al., 2023), miR-6807-5p (Wang et al., 2021), and miR-135b (Song et al., 2017) showed inhibitory effects on osteogenic/odontogenic potential at different stages. Notably, related fate-regulatory mechanisms are evident during embryonic development. During early dental morphogenesis, the spatiotemporal expression pattern of IGF-1 (Kero et al., 2015) and its target miR-15b-5p, together with signaling mediated by miR-206-3p (Neupane et al., 2020), may shape the initial developmental trajectory of odontogenic cells.

### 6.3 MiRNA regulation of angiogenesis, lineage plasticity, and cell survival

Beyond hard-tissue repair, restoration of the pulpal blood supply and tissue survival also depends on miRNA regulation. In angiogenesis, miR-424 has been reported to promote angiogenesis (Liu et al., 2014). Periodontitis-associated stem cells secrete extracellular vesicles rich in miR-378a to promote local vascularization by targeting Sufu and activating the Hedgehog/Gli1 signaling pathway (Zhou et al., 2021). Vesicles from hypoxia-conditioned stem cells further enhanced this vascular repair effect (Liu et al., 2022).

For cell survival and proliferation, miR-584 knockdown can promote proliferation (Tian et al., 2020). Downregulation of miR-224-5p promotes cell migration and proliferation (Ke et al., 2019), while its expression protects dental pulp stem cells from apoptosis by targeting Rac1 (Qiao et al., 2020). Evidence from non-pulp repair models was retained only as indirect support for DPSC-derived vesicle cytoprotection (Shi et al., 2023). These non-pulp studies provide context for DPSC plasticity because intrinsic miRNA programs define basal stem cell characteristics (Vasanthan et al., 2015) and totipotency boundaries (Pinheiro et al., 2026; Tan and Dai, 2017), and influence soft-tissue differentiation. Other studies illustrate DPSC plasticity but do not directly address pulpitis mechanisms. For example, Xie and Shen reported that miR-139-5p promotes skeletal myogenic differentiation of human adult DPSCs through the Wnt/β-catenin pathway (Xie and Shen, 2018). Liu et al. showed that pleiotrophin modulates the chondrogenic differentiation potential of DPSCs under normal and inflammatory microenvironments (Liu C. et al., 2024). Pan et al. further demonstrated that miR-20a-5p targets SMAD6 and inhibits chondrogenic differentiation of hDPSCs (Pan et al., 2023). These studies indicate that DPSC fate is shaped by lineage-specific post-transcriptional programs, but they are not presented as direct evidence for pulpitis pathogenesis.

### 6.4 Aging, biomaterial interfaces and biomimetic tissue engineering

Cellular repair capacity varies with donor age and matrix context. Transcriptomic comparisons revealed distinct miRNA expression profiles between the dental pulp of older and younger donors (Wang et al., 2015). Analyses of mesenchymal stromal cells identified tissue-specific miRNA changes associated with age-related declines in repair potential (Iezzi et al., 2021).

Tissue engineering approaches that combine biomaterials with targeted delivery may overcome limitations of cell-based approaches. For gene activation at inorganic interfaces, Portland cement (Wang et al., 2018), unwashed absorbable sandblasting medium (NWRBM) titanium surface (Gardin et al., 2016), and equine bone substitutes (Tumedei et al., 2022) have been shown to enhance osteogenic attachment and differentiation of stem cells at material interfaces by upregulating specific miRNAs such as miR-146a. In more advanced biomimetic designs, researchers immobilized exosomes carrying osteogenic miRNAs on titanium scaffolds to accelerate bone integration (Zhang et al., 2024). Other studies isolated “lineage-specific exosomes” from specific differentiation stages. These biomimetic vesicles can cross cell boundaries and induce naïve stem cells to differentiate toward the dentin lineage (Hu et al., 2019), providing a potential cell-free strategy for future dental pulp tissue engineering.

## 7 Conclusions and future directions

A two-axis framework helps organize miRNA functions in pulpitis: an immune-regulatory axis that controls inflammatory amplification and resolution, and a repair-competence axis that shapes DPSC survival, angiogenesis and osteogenic/odontogenic differentiation. This framework clarifies why the same miRNA network may support host defense in one context but impair regeneration in another.

Pulpitis progression and repair are shaped by interacting immune, epigenetic and regenerative signaling networks, in which miRNAs and miRNA-linked ceRNA circuits coordinate context-dependent responses. miRNA regulators therefore remain candidate therapeutic targets for pulpitis and endodontic tissue repair (Palideh et al., 2023). For translation, extracellular vesicles (EVs) are being investigated as tools for diagnosis and therapeutic delivery because of their natural biocompatibility and capacity to carry regulatory nucleic acids (Andjus et al., 2020).

### 7.1 Engineered vesicles and nanoframes for miRNA delivery

A central bottleneck for clinical translation is protecting fragile non-coding RNAs while achieving targeted, durable delivery within the pulp cavity. Modifying the surface or contents of vesicles through genetic engineering to develop engineered exosome therapies may enhance tissue-specific accumulation and efficacy of noncoding RNA cargo, which could address limitations of direct stem cell transplantation, including low survival and immune rejection (Saikia and Dhanushkodi, 2024). One proposed strategy uses mesenchymal stem cells that overexpress anti-inflammatory or differentiation-promoting miRNAs (such as miR-34a) to actively secrete exosomes enriched in the selected miRNA has been proposed as a potentially scalable drug delivery strategy (Vakhshiteh et al., 2021).

In addition to biogenic vectors, advances in nanochemistry have expanded options for direct miRNA delivery. For example, chemically modified, self-assembling DNA tetrahedral frameworks can load and deliver miRNAs, creating a highly stable, nuclease-resistant, and cell-penetrating nucleic acid nanocarrier platform for pulp regeneration (Wei et al., 2024). For complex dental defects, immobilization of exosomes derived from dental pulp stem cells onto inorganic biomaterials such as titanium scaffolds may enhance the biological activity of the material interface and accelerate the vascular remodeling and functional integration of local tissues (Zhang et al., 2024).

For pulpitis, therapeutic feasibility will depend on local retention within the confined pulp cavity, protection from nuclease degradation, control of dose and release kinetics, and the avoidance of off-target immune activation. These challenges are especially important because vital-pulp therapy must preserve remaining healthy tissue while suppressing inflammation and supporting repair.

### 7.2 Cell-source expansion and microenvironmental preconditioning

Scalable cell-free therapy requires a practical source of regenerative vesicles. The traditional view holds that only highly purified stem cell subpopulations can be used for regenerative therapy, but recent transcriptomic studies indicate that even unfiltered, fibroblast-dominated dental pulp cell populations secrete exosomes with mesenchymal stem cell (MSC)-like molecular signatures and distinct miRNA-mRNA regulatory axes, which could expand the donor-cell pool for future therapeutic vesicle production (Yoshida et al., 2026).

*In vitro* preconditioning with disease-relevant stressors can also alter the reparative properties of vesicles. For example, small extracellular vesicles derived from dental pulp stem cells under hypoxic preconditioning outperformed normoxic controls in reported models by inhibiting osteoclast overactivation, reducing inflammatory osteolysis, and promoting deep bone defect repair (Tian J. et al., 2023). Systemic radiation-injury models provide only indirect evidence for vesicle biodistribution and cytoprotection. In these animal models, DPSC-derived vesicles reduced hematopoietic damage after whole-body radiation (Kong et al., 2021).

### 7.3 Clinical translation: donor aging and heterogeneity control

The preceding sections show that miRNA-based translation depends on three linked variables: cargo selection, delivery platform and donor cell state. Donor heterogeneity must therefore be controlled before cell-free non-coding RNA therapies can be implemented clinically. Comparative analyses of microRNA expression profiles indicate that the tissue origin of mesenchymal stromal cells and donor senescence are important determinants of the biological characteristics of these cells and their secretome (Iezzi et al., 2021). Available *in vitro* and *in vivo* data indicate that the proangiogenic and osteoinductive activity of dental pulp stem cell-derived exosomes may decline as donor age increases (Brunello et al., 2022).

Future endodontic research should clarify miRNA interaction networks and establish rigorous donor-screening standards, pretreatment specifications and vesicle/miRNA quality-control systems. These standards are needed before miRNA-based pulp-regeneration strategies can be tested for safety, batch consistency and predictable efficacy in clinical applications.
